# Trans-bronchial lung cryobiopsy in patients at high-risk of complications

**DOI:** 10.1186/s12890-021-01503-9

**Published:** 2021-04-26

**Authors:** Benjamin Bondue, Pascal Schlossmacher, Nathalie Allou, Virgile Gazaille, Olivier Taton, Pierre Alain Gevenois, Frederic Vandergheynst, Myriam Remmelink, Dimitri Leduc

**Affiliations:** 1 Department of Pneumology, Hôpital Erasme, Université libre de Bruxelles, 808 route de Lennik, 1070 Brussels, Belgium; 2 Department of Pneumology, University Hospital of La Reunion, Saint Denis, France; 3 Department of Radiology, Hôpital Erasme, Université libre de Bruxelles, Brussels, Belgium; 4 Department of Internal Medicine, Hôpital Erasme, Université libre de Bruxelles, Brussels, Belgium; 5 Department of Pathology, Hôpital Erasme, Université libre de Bruxelles, Brussels, Belgium

**Keywords:** Surgical lung biopsy, Trans-bronchial lung cryobiopsy, Cryobiopsy, Interstitial lung disease, Diffuse parenchymal lung disease, SLB, TBLC, ILD, Pulmonary fibrosis

## Abstract

**Background:**

The surgical lung biopsy (SLB) is the recommended sampling technique when the pathological analysis of the lung is required in the work-up of an interstitial lung disease (ILD) but trans-bronchial lung cryobiopsy (TBLC) is increasingly recognized as an alternative approach. As TBLCs have lower mortality and morbidity risks than SLB, this study aimed to investigate the safety of TBLCs in patients at higher risk of complications and for whom SLB was not considered as an alternative.

**Method:**

This prospective study was conducted in two hospitals in which TBLCs were performed in patients with body mass index (BMI) > 35, and/or older than 75 years, and/or with severely impaired lung function (FVC < 50% or DLCO < 30%), and/or systolic pulmonary artery pressure > 45 mmHg, and/or a clinically significant cardiac disease. Patients with any of these risk factors constituted the high-risk group. Clinical outcomes were compared with those obtained in patients without these risk factors (low-risk group).

**Results:**

Ninety-six patients were included between April 2015 and April 2020, respectively 38 and 58 in the high-risk or the low-risk group. No statistically significant difference was observed between both groups in terms of severity and rate of bleeding, pneumothorax, or duration of hospital stay (*p* value ranging from 0.419 to 0.914).

**Conclusion:**

This preliminary study on a limited number of patients suggests that TBLC appears safe in those in whom lung biopsy is at high-risk of complications according to their age, BMI, lung impairment, and cardiac comorbidities.

**Supplementary Information:**

The online version contains supplementary material available at 10.1186/s12890-021-01503-9.

## Introduction

Interstitial lung diseases (ILDs) are a heterogeneous group of diseases with variable amounts of fibrosis and inflammation. For prognostic and therapeutic purposes, a precise diagnosis is required. This diagnosis is reached during multidisciplinary discussion (MDD) of the clinical, radiological, and pathological data [1, 2]. If a lung specimen is required, surgical lung biopsy (SLB) is recommended [1, 2] but is invasive, requests pleural drainage, and is associated with a mortality rate ranging from 2.0 to 3.6% [3–8]. The risk of complications is higher if SLB is performed in non-elective patients, during acute exacerbation, in immunocompromised patients, or in those with lower lung function or diffusion capacity, or with pulmonary hypertension [3, 8–10].

Trans-bronchial lung cryobiopsy (TBLC) is increasingly recognized as an alternative approach in ILDs [6, 11–14], the information provided being similar to that from SLB in the setting of MDD [15–17]. The advantages of TBLC are its lower mortality and morbidity rates, reduced hospital stay with subsequent potential cost saving [6, 12, 18]. A recent meta-analysis showed a pooled diagnostic yield of 84% for TBLC with moderate to severe endobronchial bleeding and post-procedural pneumothorax rates respectively of 4.9% (95% CI; 2.2–10.7%) and 9.5% (95% CI; 5.9–14.9%) [19]. However, these data were obtained from studies with variable exclusion criteria and often exclusion of the most severely affected patients, in particular those with forced vital capacity (FVC) < 50% and/or diffusing capacity for carbon monoxide (DLCO) < 35% and/or significant pulmonary hypertension, generally considered as relative contra-indications to TBLC [13, 20]. However, very limited data support increased risks of TBLC in patients with altered lung function and/or low diffusing capacity [21, 22] and, it is unclear whether TBLC could be safely performed in those with increased systolic pulmonary artery pressure, obesity, and/or older than 75 years. The aim of the present study was therefore to investigate the safety of TBLC in patients at risk of complications according to their age, body mass index (BMI), lung impairment, and/or clinically significant cardiac comorbidities. As they are generally not eligible for SLB, the evaluation of the complication rate of TBLC is particularly of interest in these patients.

## Material and methods

### Study design

A prospective observational study was conducted between April 2015 and April 2020 in Erasme university hospital (Brussels, Belgium) and Centre Hospitalier Universitaire of La Reunion (Saint Denis, France). The study protocol was approved by the leading ethical committee of the Erasme university hospital (Ref. Nr: P2015/192). Written informed consent was obtained from each patient. All procedures were carried out in accordance with relevant guidelines and regulations. Adult patients (older than 18 years) were included in our study group if a lung biopsy was decided in MDD according to the most recent guidelines [1]. Only elective procedures were considered for inclusion. All anticoagulant or antiplatelet therapies (including N-acetylsalicylic acid) were stopped before the procedure according to the appropriate drug-specific interval and following recommendations for high-risk procedures [23]. Patients with an excessive risk of bleeding (known coagulopathy, platelet count < 100,000 per µl, prothrombin time international normalized ratio—INR > 1.5, activated partial thromboplastin time—APTT > 35, or inability to stop anticoagulant therapy or antiplatelet therapy) and those with an excessive risk of anesthesia as judged by the investigators were not considered for inclusion. This includes patients with a significant hypoxemia (PaO_2_ < 55 mmHg at room air), hypercapnia (PaCO_2_ > 50 mmHg), or cardiac comorbidities (acute heart failure (< 1 month), instable angina pectoris, recent coronary artery disease (< 3 months), or any cardiac disease with evidence of pulmonary edema).

Patients were further categorized in a high-risk group according to the presence of any of the following criteria: ≥ 75-year-old, BMI ≥ 35, systolic pulmonary arterial pressure (sPAP) estimated by echocardiography ≥ 45 mmHg, FVC < 50% or DLCO < 30%, or significant cardiac comorbidities with reduced heart ejection fraction. These cut-offs were determined either arbitrary (age), or based on our clinical practice at the time of the initiation of this study [12].

In our clinical practice, patients who have any of these risk factors were also considered by the MDD has not eligible for SLB.

Indications and results of TBLC were multidisciplinary discussed as recommended in suspected idiopathic pulmonary fibrosis (IPF) [1]. At least one chest physician, one pathologist, one thoracic radiologist, one specialist in internal medicine or rheumatology participated to the MDD conducted in each of both participating hospitals.

### Bronchoscopy and TBLC

Interventions were performed under general anesthesia, intubation with a rigid bronchoscope, in a bronchoscopy suite with standard monitoring and sedation procedures. For TBLC, participating hospitals used flexible cryoprobe of 2.4 mm or 1.9 mm in diameter (ERBE, Germany). The cryoprobe operated using the Joule–Thomson effect, in which compressed gas (CO_2_) underwent an adiabatic expansion and rapidly cools the probe tip to − 45 °C within several seconds. The biopsies were obtained by insertion of the cryoprobe through the working channel of a flexible bronchoscope placed into the rigid bronchoscope. Biopsies were performed in the most affected areas excepted where honeycomb changes predominated. We attempted to obtain four samples from two different segments of the same lobe. For each biopsy, the cryoprobe was pushed under fluoroscopic guidance to the distal parenchyma and withdrawn of one-two cm from the thoracic wall. Once in position, the probe was cooled for five to six seconds, then the probe and the bronchoscope were removed *en-bloc* out of the airway, and the frozen specimen was thawed first in saline at room temperature and afterwards transferred to formalin for fixation. To control potential severe bleeding, a Fogarty balloon was systematically placed in the lobar bronchus close to the sampled segment, and inflated immediately after biopsy. The bleeding was scored 0 if absent, 1 (mild) if stopped with aspiration only and/or insufflation of the Fogarty balloon less than five minutes, 2 (moderate) if cold saline was used to control the bleeding and/or the Fogarty balloon needed to be inflated more than five minutes, and 3 (severe) if any of the following treatments was required: embolization, selective bronchial intubation, transfusion, admission in intensive care unit (ICU), or resulting in death or prolonged hospital stay. Within three hours after the procedure, a chest radiograph was obtained to look for pneumothorax. All patients stayed at hospital for one night after the procedure for monitoring in order to identify relapse of bleeding and subacute pneumothorax.

### Biopsy specimens

Biopsy specimens were fixed in 10% formalin and embedded in paraffin. Hematoxylin and eosin as well as Masson's Trichrome, Giemsa staining were performed as well as immunostaining against pancytokeratins.

### Statistical analysis

The study was powered to detect clinically significant increase risk of pneumothorax (three or more-fold) and severe bleeding (five or more-fold). The minimal size of the study population (68 patients) was determined using the BiostaTGV online application by imposing an alpha error of 5% and a power (1-β) of 80%. Primary endpoints were the rate and severity of bleeding, the rate of pneumothorax, and the duration of hospital stay after the procedure.

According to the D’Agostino & Pearson test used to challenge the normality of the distributions; comparisons between two groups were tested by unpaired Student t tests or Mann–Whitney tests. Proportions were compared using the Chi^2^ test. Statistical analyses were performed using GraphPad Prism 6 (GraphPad Software, La Jolla, CA). For all tests, a *P*-value of less than 0.05 was considered statistically significant.

## Results

Ninety-six patients were included. Their main characteristics are summarized in Table 1. Thirty-eight patients were categorized in the high-risk group (15 patients with BMI ≥ 35, 15 patients with severe pulmonary impairment, four patients older than 74 years, four patients with sPAP ≥ 45 mmHg and three patients with clinically significant ischemic heart disease with altered left ventricular ejection fraction rate). Three patients cumulated two risk factors (two patients with both lung function impairment and elevated sPAP, and one patient with both lung function impairment and BMI ≥ 35).

**Table 1:** Characteristics of the patients included in the study who had TBLCs for the work-up of ILD

		All (n = 96)	Low-Risk (n = 58)	High-Risk (n = 38)	*p* value
*Patient characteristics*					
Gender	Male, n (%)	53 (55)	30 (52)	23 (61)	0.396
Age, years	Mean (SD)	63 (9)	62 (9)	65 (10)	0.165
Smoking history	Current, n (%)	12 (13)	10 (17)	2 (5)	0.083
	Former, n (%)	50 (52)	26 (45)	24 (63)	0.079
	Never, n (%)	34 (35)	22 (38)	12 (32)	0.524
BMI	Mean (SD)	28 (5)	27 (4)	31 (6)	< 0.001
FVC, % predicted value	Mean (SD)	72 (22)	79 (19)	61 (23)	< 0.001
DLCO, % predicted value	Mean (SD)	51 (16)	54 (15)	45 (16)	0.006
sPAP	Mean (SD)	30 (9)	27 (6)	33 (12)	0.005
*Thin-section CT pattern*					
UIP	n (%)	1 (1)	1 (2)	0 (0)	0.419
Probable UIP	n (%)	12 (13)	6 (10)	6 (16)	0.430
Indeterminate for UIP	n (%)	33 (34)	21 (36)	12 (32)	0.641
Alternative diagnosis	n (%)	50 (52)	30 (52)	20 (52)	0.931
*TBLC*					
Diameter of the Cryoprobe	1.9 mm, n (%)	8 (8)	3 (5)	5 (13)	0.166
	2.4 mm, n (%)	88 (92)	55 (95)	33 (87)	0.166
Number of specimens/patient	Mean (SD)	4 (1)	4 (1)	4 (1)	0.148
Size of specimens (mm^2^)	By specimen Mean (SD)	21 (7)	20 (7)	22 (8)	0.153
	By patient Mean (SD)	78 (24)	74 (24)	81 (24)	0.138
*Final MDD diagnosis*					
IPF	n (%)	35 (36)	19 (33)	16 (42)	0.352
HP	n (%)	34 (35)	20 (34)	14 (37)	0.813
Other	n (%)	27 (28)	19 (33)	8 (21)	0.695

Patients were further categorized in low- and high-risk groups. Low- and high-risk groups were compared with unpaired Student t tests or the Chi^2^ tests for proportions. A *p*-value of less than 0.05 was considered statistically significant

Definition of Abbreviations: BMI: body mass index; CT: computed tomography; DLCO: diffusing capacity for carbon monoxide; FVC: forced vital capacity; MDD: multidisciplinary discussion; SD: standard deviation; sPAP: systolic pulmonary artery pressure; TBLC: transbronchial lung cryobiopsy; UIP: usual interstitial pneumonia

No statistically significant difference was observed between low-risk and high-risk groups in terms of gender, smoking history, radiological patterns, and size of the lung specimen (Table 1). As expected, statistically significant differences were observed for BMI, sPAP, FVC, and DLCO, but not for age. In the whole study group, the final diagnosis reached at MDD was mainly IPF in 35 patients and hypersensitivity pneumonitis (HP) in 34 patients without statistically significant differences between groups. Less frequent diagnoses are listed in the Additional file 1: Table S1.

**Fig. 1:**
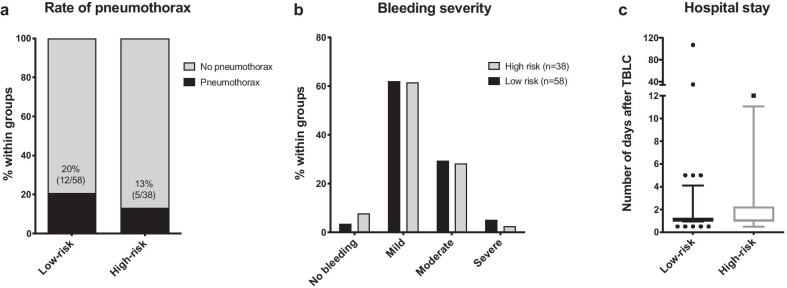
Comparison between low- and high-risk groups for pneumothorax (**a**), bleeding severity (**b**), and the hospital stay duration after TBLC (**c**)

Regarding safety, no statistically significant difference between low- and high-risk groups was observed in terms of bleeding or pneumothorax (Fig. 1 and Table 2). The incidence of pneumothorax was 20% and 13% in the low- and the high-risk groups respectively (*p* = 0.419). Almost 50% of them required a chest drainage, without difference between groups (*p* = 0.687). Subgroup analysis were performed to evaluate the incidence of pneumothorax within 15 patients with BMI ≥ 35 or 15 other patients with FVC < 50% or DLCO < 30% and those in the low-risk group. No statistically significant difference was observed between these subgroups, the incidence of pneumothorax being 7% for patients with BMI ≥ 35 and for patients with FVC < 50% or DLCO < 30% compared to the rate of 20% in the low-risk group; *p* = 0.206 and 0.316, respectively) (Additional file 2: Figure S1). Subgroup analysis was not performed for patients belonging to the high-risk group according to age, sPAP, or due to cardiac comorbidities as these subgroups were insufficiently powered to draw statistically significant conclusion.

**Table 2:** Comparison of the safety data between low- and high-risk groups for TBLC

		Low-risk (n = 58)	High-risk (n = 38)	*p* value
*Bleeding n (%)*				
No	n (%)	2 (3)	3 (8)	0.338
Mild	n (%)	36 (62)	24 (61)	0.914
Moderate	n (%)	17 (30)	11 (28)	0.969
Severe	n (%)	3 (5)	1 (3)	0.542
Pneumothorax	n (%)	12 (20)	5 (13)	0.419
Chest tube	n (%)	6 (10)	3 (8)	0.687
Other complications	n	AE-ILD: 1 Empyema: 1 Seizure: 1	Death: 1	
Hospital Stay after TBLC (day)	Median (range)	1 (1–107)	1 (1–12)	0.675

Groups were compared with unpaired Student t tests (parametric data), Mann Whitney (non-parametric data), or the Chi^2^ test (proportions). A *P*-value of less than 0.05 was considered statistically significant

Definition of Abbreviation: AE-ILD = acute exacerbation of interstitial lung disease; TBLC: transbronchial lung cryobiopsy

**Fig. 2:**
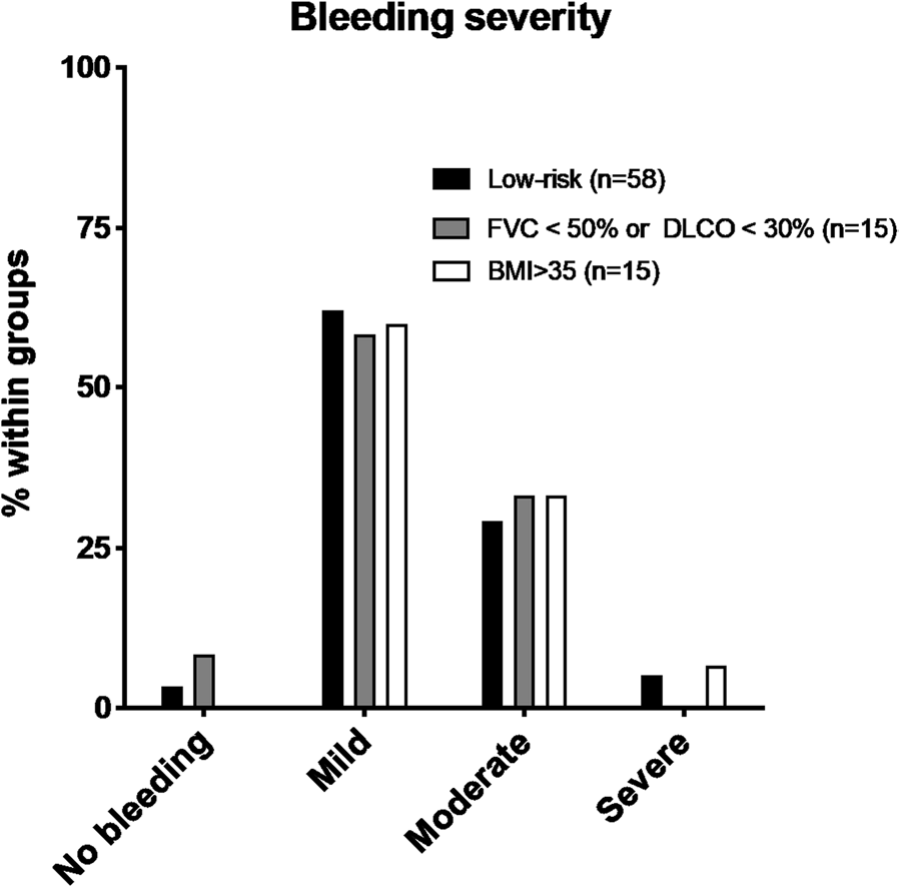
The rate of bleeding and its severity scores were compared between the low-risk group and the high-risk group according to impaired lung function or high BMI. BMI: body mass index; DLCO: diffusing capacity for carbon monoxide; FVC: forced vital capacity

When occurring, bleeding was mild in approximately 60% of patients (62% and 61% respectively in the low- and high-risk group) (*p* = 0.914) and severe bleeding in 5% and 3%, respectively (*p* = 0.542) (Fig. 1 and Table 2). Subgroup analysis among patients in the high-risk group for high BMI or advanced lung impairment revealed similar incidence of bleeding regardless these risk factors compared to patients in the low-risk group (Fig. 2). No subgroup analysis was performed for patients with an age over 74, a sPAP over 45 mmHg or significant cardiac comorbidities according to their low representation (less than five patients). Taking into account a one-day monitoring after the procedure as requested by the protocol, the median hospital stay after the procedure was not longer that one day without difference between subgroups (Table 2 and Fig. 1) (*p* = 0.675). In the low-risk group, one patient experienced acute exacerbation of the underlying fibrotic disease and remained hospitalized 107 days while waiting for lung transplantation, one patient had a pneumothorax complicated by empyema, and one patient presented a seizure 24 h after the procedure. No death was recorded after the procedure in the low-risk group but one patient in the high-risk group who had a severe underlying ischemic cardiac disease died suddenly four days after the procedure, suggesting a possible acute embolic and/or coronary event (no autopsy could be performed). The overall mortality rate in our study group was 1/96 (1%). In both groups, no re-admission or delayed pneumothorax was observed. Among the three patients cumulating two risk factors, no pneumothorax were observed. No severe bleeding was also recorded in this subgroup (no bleeding in one patient; mild and moderate bleeding in the two patients).

Finally, the results from both participating hospitals were compared. The rates of men (71% *vs.* 49%, *p* = 0.040) and HP diagnosis (53% *vs.* 26%, *p* = 0.011), and the proportion of high-risk patients (64% *vs*. 29%, *p* < 0.001) were higher in one hospital than in the other. However, no statistically significant difference was observed between hospitals in terms of severity of bleeding, incidence of pneumothorax, or duration of the hospital stay (Additional file 1: Table S1).

## Discussion

This study shows that TBLC can be safely performed in patients considered at risk of complications according to the presence of any of the followings: obesity (BMI ≥ 35), suspected pulmonary arterial hypertension (sPAP ≥ 45 mmHg), advanced lung function impairment (FVC < 50% or DLCO < 30%), cardiac comorbidities with reduced heart ejection fraction, or over 74-year-old. These criteria were selected on the basis of our previous experience and clinical practice [12]. Similar cut-off value for age was also used as exclusion criteria in prospective studies evaluating the safety of SLB [5, 24]. The cut-off values for sPAP and FVC are also similar to those reported by Hetzel and colleagues as relative contra-indications for TBLC in an expert statement published after the initiation of this study [11], with a slight difference in DLCO cut-off values (30% *vs.* 35%).

Thirty-eight patients with at least one of these risk factors were identified and pooled into the high-risk group. The safety data obtained when performing TBLC in these patients did not showed a higher rate of clinically significant complications, mortality, or prolonged hospital stay as compared to those without these risk factors. In the high-risk group, one patient died four days after the procedure possibility because of an acute embolic and/or coronary event whereas one patient in the low-risk group presented an acute exacerbation of his ILD and survived thanks to lung transplantation. Altogether, these major adverse events, even infrequent, highlight that TBLC remains a possible life threatening procedure that should be performed only after MDD. Moreover, these results were obtained by trained endoscopy teams who used systematically a Fogarty balloon to control possible bleeding and monitored the patient at hospital for 24 h after the procedure.

The observation that TBLC can be performed without major increase of complications rate in patients globally estimated at risk of complications is important in clinical practice. Although the criteria used to define the high-risk group are rather those defined for TBLC, patients belonging to this high-risk group were also not considered as eligible for a SLB by our MDD. Advanced lung function impairment is indeed usually considered as a contra-indication for SLB, elderly patients (older than 70 or 75 years) are generally excluded from prospective studies evaluating the safety of SLB and the mean age in large retrospective study was around 60 years [3, 5, 8–10, 24]. Moreover, limited data highlight the role of high BMI or pulmonary hypertension as risks factors of complications following SLB [8] or more generally thoracic surgery [25]. Consequently, a SLB was not been considered as an alternative. Therefore, the possibility to perform TBLC without major increase in the complication rate is of interest as the resulting pathological data obtained by the TBLC was useful in the context of the multidisciplinary diagnosis of the underlying interstitial disease [15, 17].

In patients with advanced lung function impairment, the risk of complications induced by TBLC was in line with those reported by Ravaglia et al*.* in a retrospective study. In this study, 297 patients who had TBLC for the diagnosis of ILD were compared to 150 patients who had biopsy by video-assisted thoracoscopic surgery (VATS). This study showed that the severity of the lung function impairment was not related to the occurrence of complications in patients who had TBLC whereas the lung function was more compromised in those who developed subsequent complications after VATS biopsy [6]. At the opposite, our results contrast with other and limited data supporting an increased risk of TBLC in patients with severely altered lung function or low diffusing capacity [21, 22]. Aburto et al*.* reported increased risk of admission to hospital and more frequent complications in patients with lower FVC or DLCO values [21]. Ravaglia et al*.* reported a higher rate of pneumothorax among patients with lower FVC or DLCO values [22]. These relationships were based on multivariable analysis performed from large cohorts (257 patients analyzed by Aburto et al*.* and 699 patients by Ravaglia et al*.*). Interestingly, our results were prospectively obtained and suggest that the higher risk for patients with impaired lung function as observed in large retrospective cohorts might be statistically but perhaps not clinically significant. However, with only 15 patients, this subgroup of patients was too small to allow us to draw definite conclusions.

Regarding obese patients, no increased risk of complications or admission was observed as similarly observed by Aburto et al*.* [21]. We observed a relatively high rate (18%) of pneumothorax. This observation could be explained by several known risk factors of pneumothorax in our study group: a high proportion of histological UIP pattern (37%); the use of large cryoprobes of 2.4 mm of diameter in a vast majority of patients (92%); the relatively high number of samples performed (four samples in all patients) as reported by Ravaglia and colleagues (increased risk of pneumothorax from 11.4% with one or two samples *to* 21.6% with more than three samples) [22].

Our study has limitations. First, an important limitation is its relatively small size of the high-risk group. Indeed, the study was powered only to detect major increase in the risk of complications such as pneumothorax or severe bleeding. Second, the high-risk group is heterogeneous. Indeed, a majority of patients in this group had advanced disease (FVC < 50% or DLCO < 30%) or high BMI while a minority of them were older than 74 years or had cardiac co-morbidities (including suspected pulmonary hypertension). Even if no major safety concerns rise from this trial, further studies with more numerous patients are required before drawing definite recommendations, in particular for confirming small increased risks of bleeding, or pneumothorax, and for evaluating risks associated with individual factors. Indeed, our study was only designed to assess the global risk in high-risk patients and was not powered to determine risks related to individual factors such as age, obesity, cardiac comorbidities or pulmonary arterial hypertension.

In conclusion, TBLC could be performed without substantial increase of risks in patients more at risk of complications because of the health condition tested. The safety of TBLC in these patients is of interest as SLB is generally not considered in such patients. As the number of patients in this trial remains low, further studies are required to definitely establish the safety of TBLC in these patients.

## Supplementary Information

**Additional file 1: Table S1.** Clinical characteristics, lung function, TBLC diagnoses, complications, and hospital stay duration in the whole study group and in both participating hospitals. Both hospitals were compared with unpaired Student t tests or the Chi2 test (proportions). A *P*-value of less than 0.05 was considered statistically significant.

**Additional file 2: Figure S1.** Rate of pneumothorax among patients with an advanced lung impairment (FVC<50% or DLCO <30%, n=15) (**A**), among obese patients (BMI > 35, n=15) (**B**) and patients in the low-risk group (n=58).

## Data Availability

The datasets used and/or analyzed during the current study are available from the corresponding author on reasonable request.

## Abbreviations

AE-ILD: Acute exacerbation of interstitial lung disease
APTT: Activated partial thromboplastin time
BMI: Body mass index
CHD: Chronic heart disease
CT: Computed tomography
DLCO: Diffusing capacity for carbon monoxide
FVC: Forced vital capacity
HP: Hypersensitivity pneumonitis
ICU: Intensive care unit
ILD: Interstitial lung disease
INR: Prothrombin time international normalized ratio
IPF: Idiopathic pulmonary fibrosis
MDD: Multidisciplinary discussion
SLB: Surgical lung biopsy
sPAP: Systolic pulmonary artery pressure
TBLC: Transbronchial lung cryobiopsy
UIP: Usual interstitial pneumonia
VATS: Video-assisted thoracoscopic surgery
