# Supplementary figures and images for: Trans-bronchial lung cryobiopsy in patients at high-risk of complications
Source: BMC Pulm Med. 2021 Apr 26;21:135. doi: 10.1186/s12890-021-01503-9 (PMC8074461; doi:10.1186/s12890-021-01503-9)

## Slide 1
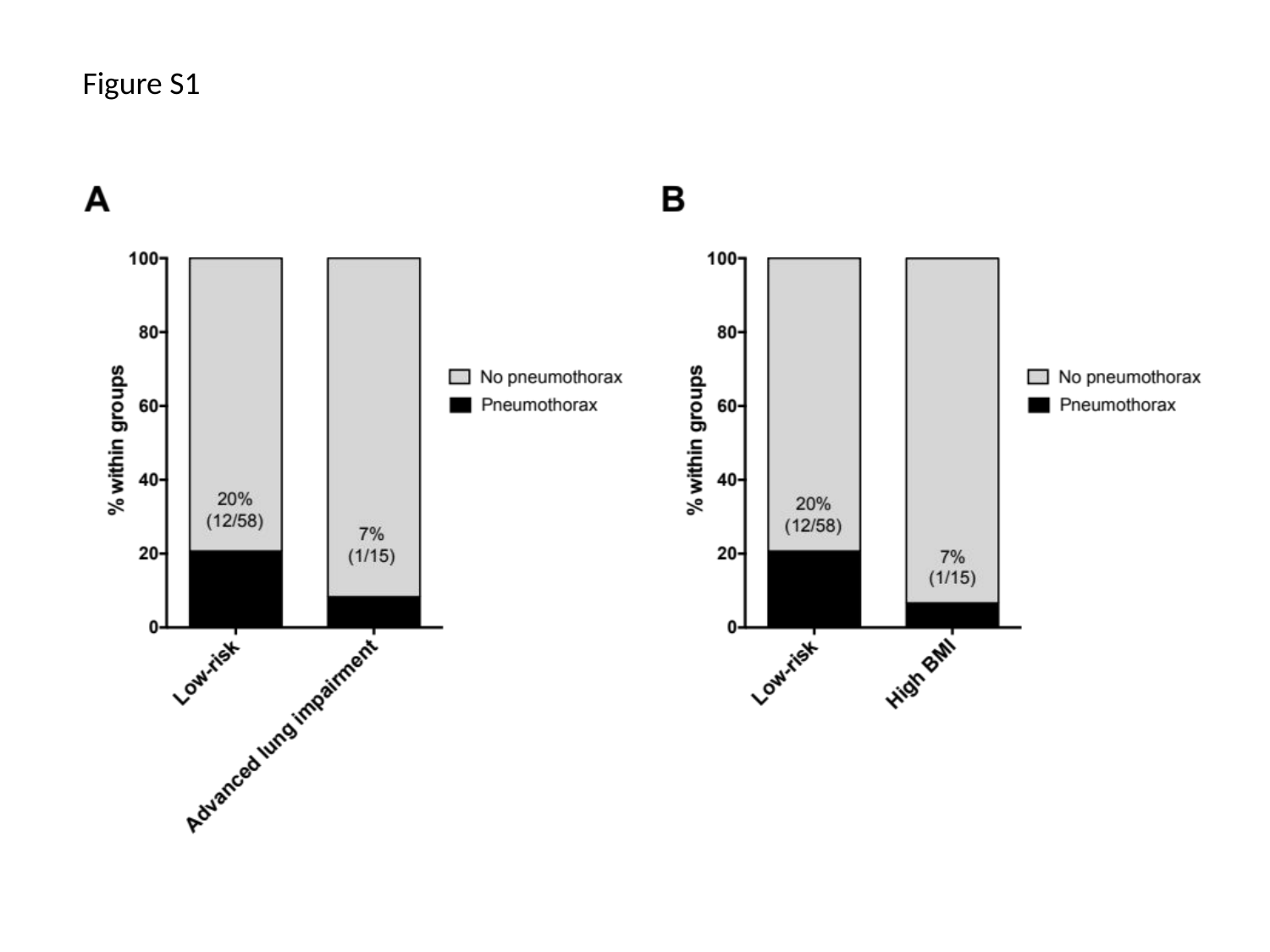

Figure S1

Supplement: Supplementary file 2 — Additional file 2: Figure S1. Rate of pneumothorax among patients with an advanced lung impairment (FVC<50% or DLCO <30%, n=15) (A), among obese patients (BMI > 35, n=15) (B) and patients in the low-risk group (n=58). [file 12890_2021_1503_MOESM2_ESM.pptx]
